# Two Decades of Experience With Chronic Mesenteric Ischaemia and Median Arcuate Ligament Syndrome in a Tertiary Referral Centre: A Parallel Longitudinal Comparative Study

**DOI:** 10.7759/cureus.20726

**Published:** 2021-12-27

**Authors:** Sherif A Sultan, Yogesh Acharya, Mohamed Mustafa, Niamh Hynes

**Affiliations:** 1 Vascular and Endovascular Surgery, Western Vascular Institute, Galway University Hospital, National University of Ireland, Galway, IRL; 2 Vascular and Endovascular Surgery, CORRIB-CÚRAM-Vascular Group, National University of Ireland, Galway, IRL; 3 General Surgery, University Hospital Ayr, AYR, GBR

**Keywords:** mesenteric ischaemia, comparative study, sympathectomy, decompression, median arcuate ligament syndrome

## Abstract

Background

Chronic mesenteric ischaemia (CMI) and median arcuate ligament syndrome (MALS) have similar clinical presentations with surgical intervention as the mainstay of treatment. However, surgical response varies and is unpredictable. Therefore, we aim to evaluate the technical and clinical success rates of selective revascularisation in older patients with CMI and younger patients with MALS undergoing arcuate ligament decompression with celiac sympathectomy.

Methods

We conducted a retrospective single-centre longitudinal comparative study of all the patients who underwent surgery for symptoms of CMI and MALS from December 2002 to 2020 at our tertiary referral vascular centre. Our primary outcome was symptom-free survival post-intervention. The secondary outcomes were perioperative mortality, technical success, and all-cause mortality at 17 years.

Results

We operated on 28 patients; 17 patients with CMI (revascularisations with bypass) and 11 with MALS (decompression and celiac sympathectomy). All (100%) patients had technical success. There was no perioperative mortality. All the MALS patients had symptom-free survival following the procedure throughout follow-up. In contrast, three patients with CMI complained of recurring abdominal pain even after one year of the surgery. However, there was no further weight loss and none of them required any intervention.

Conclusion

Stratified management of CMI with revascularisation and open surgical decompression with celiac sympathectomy in MALS are effective treatments with favourable long-term outcomes.

## Introduction

Chronic mesenteric ischaemia (CMI) is a gastrointestinal (GI) ischaemia commonly seen in older age groups [1,2]. Median arcuate ligament syndrome (MALS) is a compression syndrome (1.3/100,000 patients), mainly in young females that can cause symptoms of CMI [3,4]. CMI and MALS have similar clinical presentations, predominantly postprandial constant or intermittent abdominal pain with variable intensity and weight loss, associated with abdominal bruit [2,4]. For both CMI and MALS, intervention is the mainstay of treatment, but the response varies and is unpredictable. Therefore, we aim to evaluate the technical and clinical success rates of selective revascularisation in older patients with CMI and younger patients with MALS who underwent arcuate ligament decompression with celiac sympathectomy.

## Materials and methods

This is a retrospective single-centre parallel longitudinal comparative study of the patients who underwent surgery for symptoms of CMI and MALS from December 2002 to 2020 at our tertiary referral vascular centre. The primary outcome is symptom-free survival post-intervention. The secondary outcomes are perioperative mortality, technical success, and all-cause mortality after 17 years. Symptom-free survival is the absence of postprandial epigastric pain and weight loss after the operative procedure. Technical success is defined as recovery free of symptoms or adverse events.

Diagnostic workup

All patients underwent fasting abdominal Doppler ultrasonography (DUS), computerized tomography angiogram (CTA) of the abdominal arteries, and endoscopy. Using DUS, both preoperative and postoperative peak systolic velocity were recorded in the celiac artery (CA) and superior mesenteric artery (SMA). A positive DUS criterion for MALS was peak CA velocities of >230 cm/s, which corresponds to >70% diameter-reducing stenosis [5].

A positive CTA with a focal CA stenosis and the characteristic ‘hook-like’ appearance of the CA, best visualized on lateral projections, is diagnostic of MALS in symptomatic patients. Celiac stenosis or occlusion was predicted by evidence of retrograde flow within the hepatic arteries.

All our patients had GI endoscopies to visualize and assess upper and lower GI for mucosal perfusion or confirm any other possible pathologies without any evidence of GI pathology.

Surgical procedures

**Figure 1:**
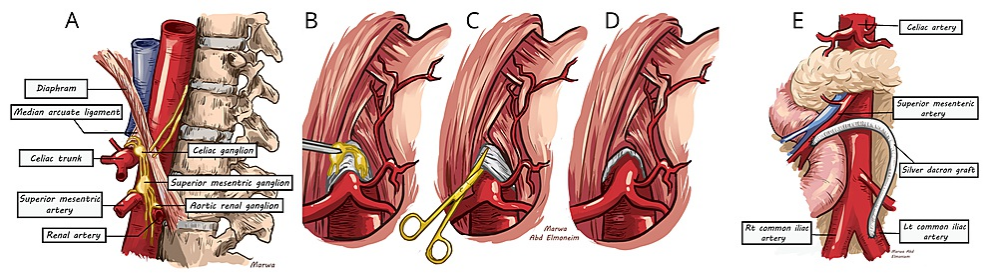
(A, B, C, D) Decompression and celiac sympathectomy in patients with median arcuate ligament syndrome. (E) Revascularisations with bypass (superior mesenteric artery to left common iliac artery) in patients with chronic mesenteric ischaemia. We confirm that this is an original image created by us.

All the patients underwent surgical interventions; decompression and celiac sympathectomy were performed in younger patients with MALS (Figure 1A-1D), and revascularisation was performed in older patients with CMI (Figure 1E). Histopathology was performed on all the samples of the celiac ganglion obtained during celiac sympathectomy from the MALS patients.

MALS and Celiac Sympathectomy Technique

**Figure 2:**
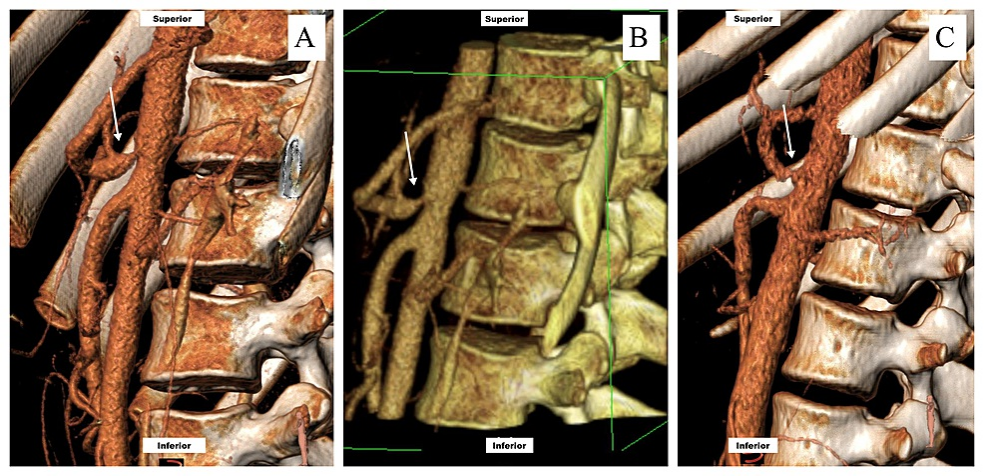
(A, B, C) Celiac stenosis (arrow) with post-stenotic dilatation in patients with median arcuate ligament syndrome.

We operated on 11 patients with MALS. A longitudinal midline epigastric incision followed by the downward retraction of the lesser curve of the stomach and the division of the left triangular ligament of the liver. The left lobe of the liver is reflected to the right. CA is palpated and located on the upper margin of the pancreas. The left gastric artery is traced to its origin, after which the whole axis is dissected free from the tough para-aortic dense autonomic nerve tissue of the CA plexus. The dissection is tedious, but freeing the whole CA to its third division is mandatory by transecting tissue overlying the anterior and medial-lateral aspects of the CA, including large nerve complexes and lymphatics. The dissection is complete when the origin of the CA and all its tributary is free of any external stricture. The MAL is divided by Metz scissors. The first few mm is the most challenging, but the ligament usually springs apart, and the CA is fully exposed. We leave post-stenotic dilatation alone to modulate over time (Figure 2A-2C).

Retrograde Celiac and SMA Revascularisation

**Figure 3:**
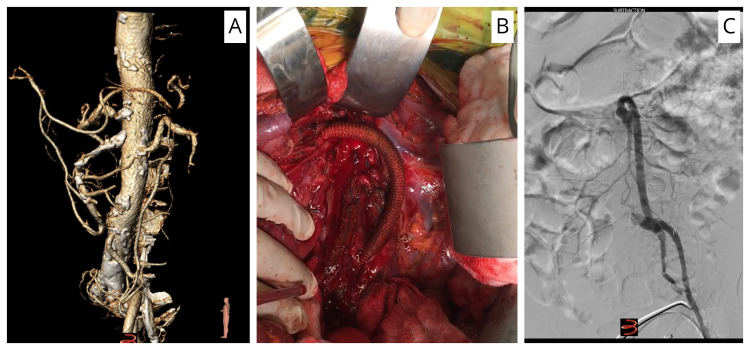
(A) A 3-dimensional reconstruction image, showing total occlusion of the celiac artery with heavy calcification, and total occlusion of the third part of the superior mesenteric artery. (B) An 8-mm silver Dacron graft in a lazy S-shape arising from the left common iliac to the third part of the superior mesenteric artery. (C) Digital subtraction angiogram, showing bypass from the left common iliac artery to the third part of the superior mesenteric artery with a silver Dacron 8 mm graft.

We operated on 17 patients with CMI. All of our bypass cases were done retrograde (Figure 3A-3B). Seven (n=7) used a common iliac artery (CIA)-based inflow, and all were performed with 8 mm silver Dacron graft to SMA (Maquet Cardiovascular®, Wayne, NJ, USA; Figure 3C). Two bypass procedures (n=2) used distal aortic based inflow to CA and SMA by bifurcated 14 mm by 7 mm silver Dacron graft. Eight revascularisations (n=8) were performed using thrombo-endarterectomy of SMA with patching and retrograde stenting using ‘Smart’ 8 mm diameter by 40 mm length stents (Cordis®, Santa Clara, CA, USA).

Two-vessel revascularisations to the SMA and CA were performed in 17.70% (n=3/17) of the cases, while single-vessel revascularisations were performed to the SMA in 82.40% (n=14/17). The grafts were configured in a lazy S shape, arising from the CIA in 41.20% (n=7/17) and the distal aorta in 11.80% (n=2/17) of the cases.

Follow-up

The patients were regularly followed up after the operative procedure, first at six weeks, then at six months, and every year after that. Each follow-up was contemplated with thorough clinical evaluations and DUS. CTA was reserved for cases that showed evidence of duplex abnormality.

Statistical analysis

Minitab (Minitab® Ltd., UK) was used for statistical analyses. We summarised continuous outcomes with mean and standard deviation (normal distribution) or median and interquartile range (non-normal distribution). The categorical outcome was summarized with percentages and proportions. For statistical significance, Wilcoxon signed-rank test and Fisher’s exact test were used. P<0.05 was taken as statistically significant.

## Results

In 17 years, we operated on 28 patients with CMI (n=17) and MALS (n=11). The baseline demographics of these patients are given in Table 1.

**Table 1:** Baseline demographics of the patients. *significant

	Total (n=28)		P-value
	Chronic mesenteric ischaemia (CMI) (n=17)	Median arcuate ligament syndrome (MALS) (n=11)	
Female	9	8	0.683
Age, mean ± standard deviation (range)	70.41 ± 8.27 (58-87)	38.40 ± 18.30 (18-67)	0.001*
Smokers	9	2	0.115
Diabetes mellitus (DM)	4	0	0.132
Chronic obstructive pulmonary disease (COPD)	2	2	1.000
Hypercholesterolemia	12	3	0.040*
Hypertension (HTN)	11	3	0.081
Ischemic heart disease (IHD)	7	0	0.023*
Chronic renal failure (CRF)	2	1	1.000

More than half of the patients (64.30%, n=18/28) presented with both postprandial pain and weight loss (CMI: 64.71%, n=11/17, and MALS: 63.64%, n=7/11; P=1.000), while 35.72% (n=10/28) had only postprandial pain (CMI: 35.30%, n=6/17, and MALS: 36.40%, n=4/11; P=1.000).

In total, 75.00% (n=21/28) patients presented with chronic onset of the symptoms (CMI: 76.50%, n=13/17, and MALS: 72.73%, n=8/11; P=1.000), while 21.43% (n=6/28) with acute on chronic (CMI: 23.53%, n=4/17, and MALS: 18.18%, n=2/11; P=1.000) and only 3.60% (n=1/28) presented with acute onset (CMI: 0%, n=0/17 and MALS: 9.10%, n=1/11, P=0.393).

Five patients (45.45%, n=5/11); four females and one male, had their diagnosis of MALS missed due to their very young age. All of them were below 25 years of age (range: 15-24). A psychiatric physician managed them after a referral from different gastroenterologists. They never had any psychiatric issues during their childhood. All have esophagogastroduodenoscopy (OGD), colonoscopies, DUS, and barium follow-through. It took between 4 months to 2 years for these patients to be referred to our practice. On arrival, they were diagnosed with the help of the DUS of the celiac axis and CTA aorta. All were cachexic in a catabolic state, with sarcopenia and malnourishment, therefore, total parenteral nutrition (TPN) was commenced gradually without worsening of the symptoms/signs. Patients with CMI had two weeks of TPN, while patients with MALS had only one week of TPN as they were relatively younger. As soon as their albumin reached above 30 g, elective surgery was performed.

Investigation

CTA was positive on 89.30% (n=25/28) patients (CMI: 100%, n=17/17, and MALS: 72.73%, n=8/11; P=0.050).

The median preoperative CA DUS flow velocity in the CMI and MALS groups was 100 cm/sec (IQR: 38 - 200) and 358 cm/sec (IQR: 316-450), respectively. Postoperatively, the median CA DUS flow velocity increased to 178 cm/sec (IQR: 100-290) in the CMI groups (P=0.248), while it decreased to 250 cm/sec (IQR: 170-290) in the MALS (P=0.010).

The median preoperative SMA DUS flow velocity in the CMI and MALS groups was 70 cm/sec (IQR: 25-198.8) and 220 cm/sec (IQR: 170-334), respectively. Six MALS patients had evidence of post-stenotic dilatation of the celiac axis. Postoperatively, the median SMA DUS flow velocity increased to 200 cm/sec (IQR: 122.5-217.5) in the CMI groups (P=0.222), while it decreased to 164 cm/sec (IQR: 145-225) in the MALS (P=0.322).

All samples from MALS were positive for neuro-fibroma on histology of the celiac ganglion following celiac sympathectomy.

Symptom-free survival

**Figure 4:**
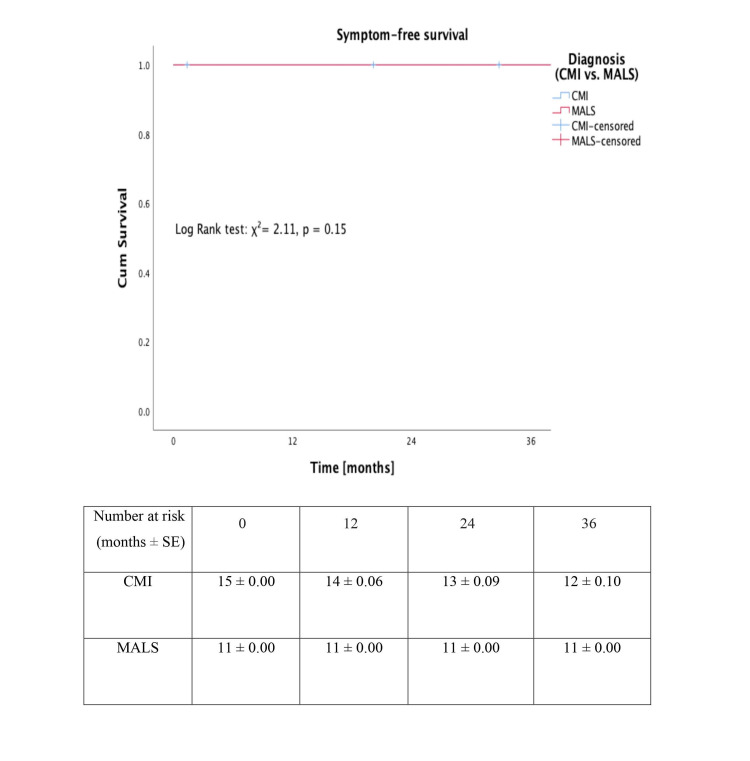
Kaplan-Meier plot showing symptom-free survival in patients with chronic mesenteric ischaemia and median arcuate ligament syndrome.

All the MALS patients had symptom-free survival following the procedure over the course of follow-up. The Kaplan-Meier plot for symptom-free survival is given in Figure 4 (log-rank test: χ²=2.11; P=0.15). Three patients with CMI complained of recurring abdominal pain even after one year of the surgery. However, there was no further weight loss and none of them required any intervention.

Perioperative and all-cause mortality

**Figure 5:**
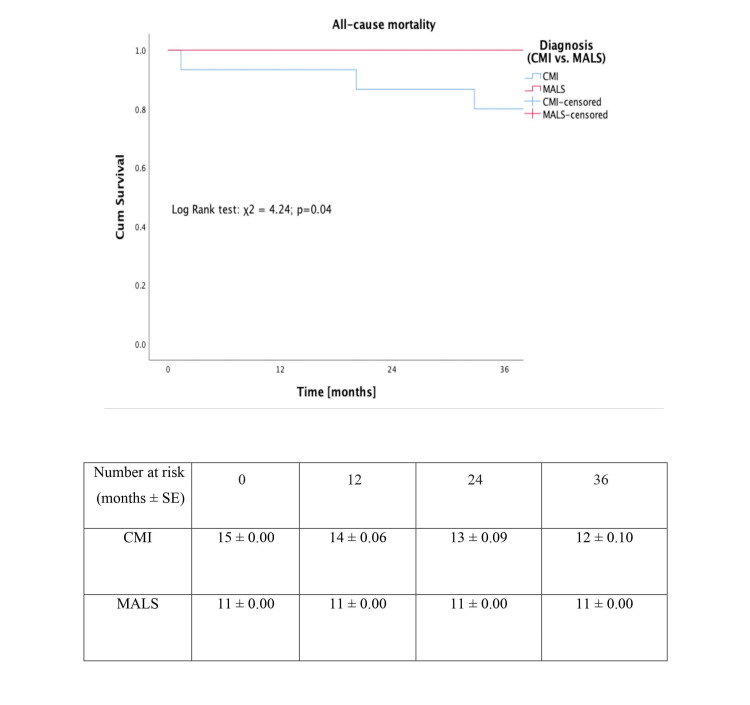
Kaplan-Meier plot showing all-cause mortality in patients with chronic mesenteric ischaemia and median arcuate ligament syndrome.

No patient had perioperative mortality. Kaplan-Meier survival plot for all-cause mortality is given in Figure 5 (log-rank test: χ²=4.24; P=0.04). None of the death was attributed to the intervention.

Technical success

**Figure 6:**
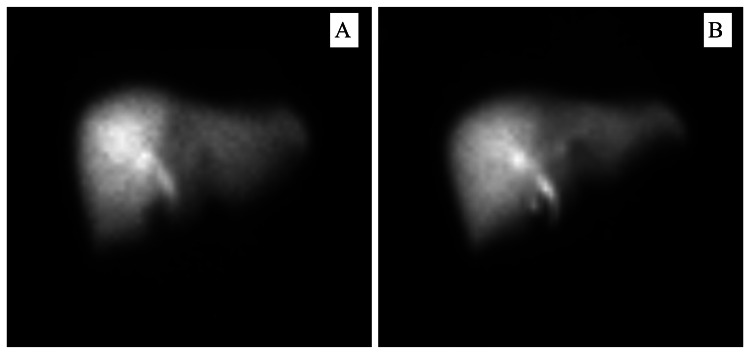
Hepatobiliary iminodiacetic acid (HIDA) scan: (A) Pre-op HIDA scan, showing an absence of biliary secretion. (B) Post-op HIDA scan, showing normal bile secretion following median arcuate ligament decompression and celiac sympathectomy.

All (100%) patients had technical success. All five young patients who were under psychiatric review had a resolution of their symptoms. Two female patients in MALS groups, mother and daughter, had MALS decompression as well as thoracic outlet syndrome (TOS) decompression, without any recurrence. Similarly, we had three patients with MALS who had no bile secretion preoperatively but drastically improved after the surgery, as shown by the hepatobiliary iminodiacetic acid (HIDA) scan (Figures 6A-6B).

Follow-up

The average length of follow-up for the CMI patients were 126 ± 85.8 months, and MALS 195 ± 28.9 months. Table 2 depicts what we have learned from our patients based on the last two decades of our experience.

**Table 2:** Comparison between chronic mesenteric ischaemia (CMI) and median arcuate ligament syndrome (MALS) based on our experience.

Characteristics	Chronic mesenteric ischaemia (CMI)	Median arcuate ligament syndrome (MALS)
Age (years)	Usually > 58	Usually < 40
Weight loss	+++	+++++
Duration of symptoms	Weeks	Months
Postprandial/epigastric pain	+++	+++++
Positional pain	No change	Decrease by leaning forward
Diarrhoea	+++++	++
Post-exertional pain	+++	+++++++
Nausea	++	++++
Abdominal bruit	+	+++++
Oesophagogastroduodenoscopy, colonoscopy	Negative	Negative
Duplex ultrasound—celiac artery (CA)	Low flow	High flow
Computerised tomography angiography—Visceral aorta	Atherosclerotic arteries	Healthy arteries
Low albumin	+++++	++
Previous psychiatry assessment	Negative	+++++
Positive psychiatry problem	Negative	Negative
Celiac artery—patency	Occluded	Compressed
Superior mesenteric artery (SMA)	Occluded	Normal
Target artery revascularisation	Single in 84%—SMA	Single in 100%—CA
Postoperative diarrhoea	Negative	++++++
Decrease in analgesia	Stopped in 76%	All stopped
Increase in body mass index	61.5%	70%
Long-term complications	+++++++	Negative
Long-term mortality	++++	Negative

## Discussion

The uncommon nature of both CMI and MALS allowed the present study to scrutinize challenging real-world scenarios to report outcomes for surgical intervention in patients with CMI and MALS.

CMI is exclusively seen in old patients with atherosclerosis as the leading cause [6]. On the other hand, MALS is usually seen in young females (mean age 40 years), causing compressive symptoms [4]. Unlike CMI, MALS is almost always restricted to the CA. Cranial origin of the CA or caudal insertion of the MAL can facilitate extrinsic compression of the proximal CA [4]. Chronic compression could lead to hyperplastic intimal changes. Histologic studies have demonstrated an abundance of smooth muscle proliferation, abnormal elastic fibres, and disorganized medial and adventitial layers that may advance to cause complete arterial occlusion in patients with MALS [4-6].

All our MALS patients had exclusive involvement of the celiac axis without thrombotic or embolic manifestations. Our patients with MALS did not have SMA involvement, contradicting the reporting by Bayat et al. [7], who attributed MALS to the compression of two visceral arteries. Furthermore, the proximity of the celiac ganglion to the compressed CA and evidence of symptomatic benefit after interventions targeting the celiac ganglion have led to postulations that neuropathic pain could be a major contributing factor in MALS [8-10].

The classical triad of CMI, postprandial abdominal pain, weight loss, and abdominal bruit, is seen only in 20% [11]. Although MALS presents similar to CMI, abdominal pain can be variable in intensity and occurs even at rest [3]. Pain may be positional, mitigated by leaning forward or drawing the knees to the chest [12,13].

Our experience spanned nearly two decades, and almost all of our MALS patients were cured after the surgical intervention compared to CMI [9]. None of our adult patients was diagnosed with any previous psychiatric illness. However, around 50% of MALS patients below 24 years of age had their diagnosis overlooked despite having a surgically modifiable condition for chronic abdominal pain. Abdominal pain linked to MALS, compared to CMI, is poorly understood due to the complex and ambiguous pathophysiologic mechanism. This unexplained mechanism has led some surgeons to hesitate in offering intervention for symptomatic MALS [14-17].

No prospective randomized controlled trials had been performed in MALS, but literature scrutiny reveals that surgery amends pain up to 85% of patients [15,18].

The diagnosis of CMI requires a high degree of clinical suspicion as there is no single gold standard test with high precision. For the screening modality of mesenteric artery stenosis, DUS is used. Gruber et al. [19] suggested that a combination of a maximum expiratory peak systolic velocity (PSV) of over 350 cm/s and a deflection angle (DA) higher than 50° in DUS seems to be a reliable indicator for MALS. CTA or magnetic resonance angiography (MRA) is the preferred imaging modality to rule out alternative abdominal pathologies, and to verify the celiac trunk location [20,21]. MALS requires dynamic imaging modalities, as the extent of stenosis depends on respiration, unlike fixed atherosclerotic pathology in CMI [12,22].

**Figure 7:**
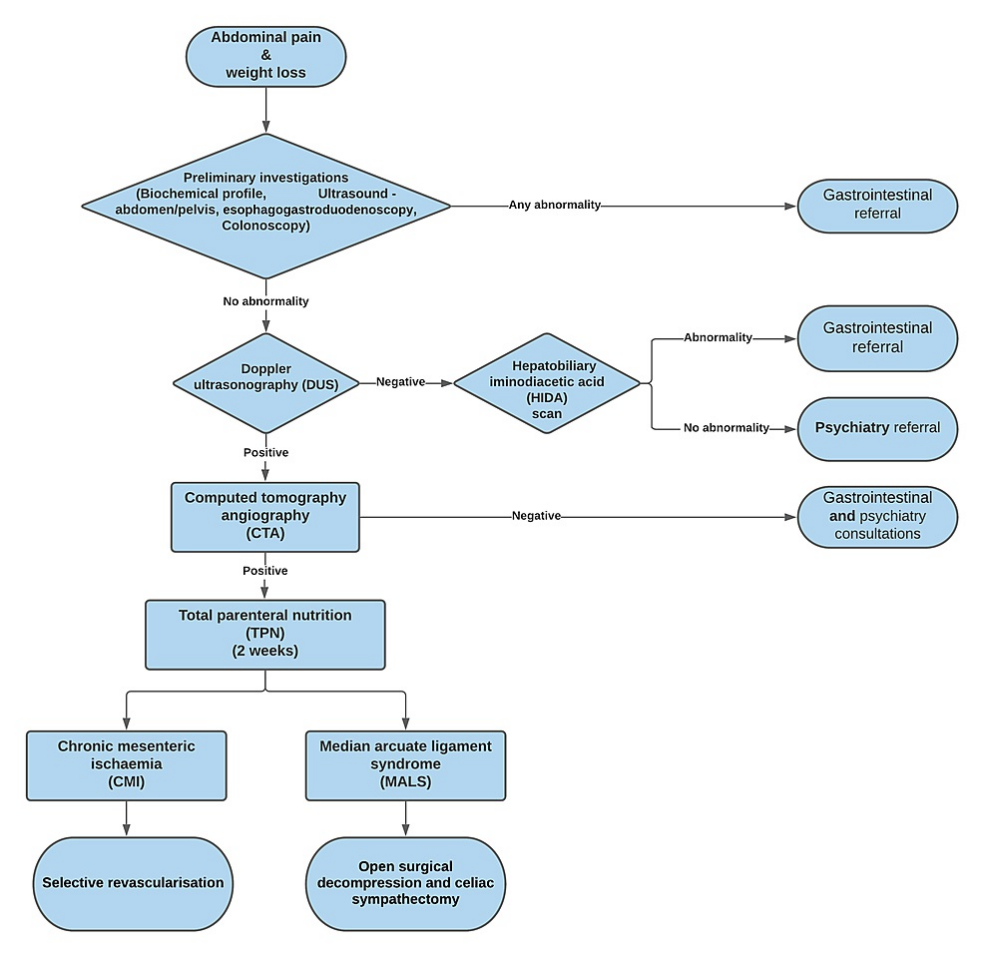
Practical therapeutic algorithm employed in patients who presented to us with abdominal pain and weight loss.

We created a checklist for our patients which include full blood count, liver enzymes, albumin, ferritin, thyroid function tests, glycated haemoglobin (HbA1C), homocysteine, abdominal pelvic ultrasound, OGD, colonoscopy, DUS of celiac and SMA before and after drinking milk and in inspiration and deep expiration, and CTA aorta. For this, we developed a simple, practical therapeutic algorithm (Figure 7). Starting with the patient referral to us with the presumptive symptoms of mesenteric ischaemia, the patient is appraised with DUS imaging by an experienced vascular scientist, where a provocative examination is accomplished during inhalation and exhalation. If positive duplex findings advocate celiac stenosis in the context of symptoms consistent with MALS, the patient is considered for CTA to delineate the CA entry and folding angle. If positive, we commence TPN for one to two weeks, aiming for albumin above 30 g/dL to improve the patient’s general ability to tolerate surgery. Those presenting with an atypical pattern of symptoms or who display equivocal duplex findings undergo more imaging with a HIDA liver scan to rule any dysfunctional biliary secretion and are consequently discussed with a gastroenterologist with a particular interest in MALS for further workup for alternative diagnoses.

Surgical management with revascularisation to relieve the occlusive atherosclerotic stenosis is necessary for CMI [20]. For MALS, intervention is aimed at addressing the pathophysiologic mechanisms with decompression of the MAL to relieve the constriction of the CA, and celiac sympathetic ganglionectomy and neurolysis to target the neuropathic component of the pain [4,23,24].

Society for vascular surgery clinical practice guidelines recommends endovascular revascularisation as the initial treatment option in CMI based on favourable early outcome [25]. However, long-term mortality was similar with both approaches, while symptoms recurrence and reintervention rates were higher with the endovascular intervention [25,26].

Delis et al. [27] contraindicated the use of angioplasty and stenting primarily in patients with MALS without the prior release of MAL and stiff para-aortic ganglia tissue with surgery. Based on our experience [9,16], any external pressure on the CA by the surrounding dense neuro-fibrous tissue compounded by the diaphragmatic critical shuttering will permit slipping of the stent or its fracture and recurrence of symptoms and signs within a short period. Furthermore, stents are crushed and displaced by the MAL if it is not fully decompressed.

We concur with the finding of Jimenez et al. [18] that post-stenotic dilatation of the celiac axis in young MALS patients can be left alone as it will modulate over time. We adopted the Reilly et al. [28] recommendations, who demonstrated that patients who underwent MAL decompression and celiac sympathectomy had lower recurrence than patients treated with ligament release alone. Furthermore, diverse pathophysiology between CMI and MALS, advocate that endovascular effectiveness in atherosclerotic mesenteric occlusive disease is not relevant to MALS, although both entities involve the CA [29,30].

Understanding CMI has taught us that endovascular options for mesenteric arteries are appropriate only for patients with short stenosis in lightly calcified vessels. However, long stenoses with extensive calcification and long occlusions with thrombus are incredibly challenging to manage by any endovascular means. Open reconstruction is a far superior option in good-risk patients [31,32]. Ryer et al. [31] reported that there were 0.9% mortality rates for open mesenteric reconstructions in good-risk patients if experienced surgeons performed such reconstructions. Nevertheless, these results are not easily reproduced in the vascular community, except in high volume centres, when the optimal treatment modality for each patient, whether open, endovascular, or a hybrid approach, can be offered [31-35].

In high-risk patients, we avoid clamping the abdominal aorta for revascularisation and always used iliac-based retrograde reconstructions with single mesenteric artery bypass [32]. Our experience mimics the findings of Kasirajan et al. [29], Ryer et al. [31], Oderich et al. [32], and Rutherford et al. [34], who had shown that isolated SMA bypasses based on the iliac artery are safe and effective. However, we learned that SMA’s excellent flow does not preclude the intensity of symptoms associated with MALS. Furthermore, we witnessed a fully patent inferior mesenteric artery in all our patients with MALS. This indicates a multifactorial aetiology at play, not only concerning the mesenteric circulation, but neurogenic and endocrine activation cascade [8-10,36-38].

All our MALS patients had extensive celiac sympathectomy to the third division of the celiac axis and with the removal of tough neuro-fibrous tissues, they became symptom-free. Neuro-fibroma of the celiac sympathetic plexus is the main culprit in recurrent abdominal pain, hence laparoscopic surgery may not allow complete neurolysis and risks a significant bleed and emergency open conversion [16]. The potential scrutiny of the neurogenic basis of MALS pain can be precluded with diagnostic celiac ganglion blockade; however, there are associated risks to these challenging interventions.

All of our MALS patients had long-term freedom from symptoms and reintervention. Our results depict the finding of Ho et al. [39] that MALS patients were more likely to respond to decompression if patients had preoperative post-exertional pain. Two of our female patients in the MALS group were mother and daughter, and both had TOS decompression through the supraclavicular approach and anterior splenectomy eight years apart. This might raise the question of genetic predisposition that requires further exploration.

Study limitations

First, we are limited with the sample size-given the uncommon nature of CMI and MALS. Furthermore, we reported CMI with MALS within the spectrum of similar vascular symptoms, despite having different pathological origins and surgical approaches. Finally, given the contrasting age differences between CMI and MALS, we cannot rule out the impact of increasing age on outcomes.

## Conclusions

Revascularisation in CMI with high atherosclerotic burden, and open surgical decompression with celiac sympathectomy in MALS are the effective treatment modalities with favourable long-term outcomes. The way forward with this kind of uncommon pathology and procedures is multicentre collaboration, preferably with a prospective study design or a registry from multiple sites. However, we firmly believe that this study will serve as a template and a reference point for future studies.
